# Systemic insecticide treatment of the canine reservoir of *Trypanosoma cruzi* induces high levels of lethality in *Triatoma infestans*, a principal vector of Chagas disease

**DOI:** 10.1186/s13071-017-2278-2

**Published:** 2017-07-19

**Authors:** Ariel Loza, Adrianna Talaga, Gladys Herbas, Ruben Jair Canaviri, Thalia Cahuasiri, Laura Luck, Alvaro Guibarra, Raquel Goncalves, Juan Antonio Pereira, Sonia A. Gomez, Albert Picado, Louisa Alexandra Messenger, Caryn Bern, Orin Courtenay

**Affiliations:** 1 Facultad de Ciencias Veterinarias, Universidad Autonóma Gabriel René Moreno (UAGRM), Santa Cruz, Bolivia; 2 University of Warwick, Gibbet Hill Campus, Coventry, CV4 7AL UK; 3 ISGlobal, Barcelona Ctr. Int. Health Res. (CRESIB), Hospital Clinic - Universitat de Barcelona, Rosello 132, 08036 Barcelona, Spain; 4 London School of Hygiene and Tropical Medicine, Keppel Street, London, WC1E 7HT UK; 5 University of California-San Francisco, 550 16th St, 94158, San Francisco, CA USA

**Keywords:** Chagas disease, *Triatoma infestans*, Dog, Bolivia, Bravecto®, NexGard®, Comfortis®, Spinosad, Fluralaner, Afoxolaner

## Abstract

**Background:**

Despite large-scale reductions in Chagas disease prevalence across Central and South America, *Trypanosoma cruzi* infection remains a considerable public health problem in the Gran Chaco region where vector-borne transmission persists. In these communities, peridomestic animals are major blood-meal sources for triatomines, and household presence of infected dogs increases *T. cruzi* transmission risk for humans. To address the pressing need for field-friendly, complementary methods to reduce triatomine infestation and interrupt *T. cruzi* transmission, this study evaluated the systemic activity of three commercial, oral, single dose insecticides Fluralaner (Bravecto®), Afoxolaner (NexGard®) and Spinosad (Comfortis®) in canine feed-through assays against *Triatoma infestans*, the principal domestic vector species in the Southern Cone of South America.

**Methods:**

Twelve healthy, outbred dogs were recruited from the Zoonosis Surveillance and Control Program in Santa Cruz, Bolivia, and randomized to three treatment groups, each containing one control and three treated dogs. Following oral drug administration, colony-reared second and third stage *T. infestans* instars were offered to feed on dogs for 30 min at 2, 7, 21, 34 and 51 days post-treatment.

**Results:**

Eighty-five per cent (768/907) of *T. infestans* successfully blood-fed during bioassays, with significantly higher proportions of bugs becoming fully-engorged when exposed to Bravecto® treated dogs (*P* < 0.001) for reasons unknown. Exposure to Bravecto® or NexGard® induced 100% triatomine mortality in fully- or semi-engorged bugs within 5 days of feeding for the entire follow-up period. The lethality effect for Comfortis® was much lower (50–70%) and declined almost entirely after 51 days. Instead Comfortis® treatment resulted in substantial morbidity; of these, 30% fully recovered whereas 53% remained morbid after 120 h, the latter subsequently unable to feed 30 days later.

**Conclusions:**

A single oral dose of Fluralaner or Afoxolaner was safe and well tolerated, producing complete triatomine mortality on treated dogs over 7.3 weeks. While both drugs were highly efficacious, more bugs exposed to Fluralaner took complete blood-meals, and experienced rapid knock-down. Coupled with its longer residual activity, Fluralaner represents an ideal insecticide for development into a complementary, operationally-feasible, community-level method of reducing triatomine infestation and potentially controlling *T. cruzi* transmission, in the Gran Chaco region.

## Background

Chagas disease, caused by the protozoan parasite *Trypanosoma cruzi*, is responsible for the highest burden of any parasitic disease in the Americas; 6 million individuals are currently infected with a further 70 million at risk [1]. *Trypanosoma cruzi* infection has a highly variable clinical course, ranging from complete absence of symptoms to severe and often fatal cardiovascular and/or gastrointestinal manifestations [2]. Despite a number of successful disease control programs in other areas of South America, intense vector-borne transmission persists in the rural Gran Chaco straddling Bolivia, Argentina and Paraguay. In the Bolivian Chaco, more than 80% of adult residents are infected and triatomine infestation of rural houses remains widespread [3]. In these endemic regions, peridomestic animals (dogs, chickens and goats, etc.) are major blood-meal sources for triatomines, and dogs are the most important *T. cruzi* reservoir, linked to high transmission risk to humans [4, 5]. Vector control is essential to prevent new disease cases. However, in the Bolivian Chaco, indoor residual spraying has failed to achieve adequate control due to poor housing conditions, suboptimal spraying efficacy and intensity, and growing insecticide resistance [3, 6]. Previous studies have explored insecticidal methods to control the reservoir of *T. cruzi* infection in canine hosts, with varying degrees of success; deltamethrin-treated dog collars reduced vector feeding success, survival, fecundity and molting rate [7, 8], while topical application of fipronil had more limited impact on triatomine mortality [9, 10]. Veterinary feed-through insecticides have shown efficacy against the vectors of leishmaniasis and malaria [11–13]. Systemic insecticides are commercially available and thus easily accessible, relatively inexpensive, easy to administer in oral formulations, long-lasting, circulate uniformly in the blood for consistent vector uptake, have good safety profiles and impart collateral benefits to treated animals (for example, simultaneous control of ecto- and endoparasites). However, to date, no work has been conducted to assess their effect on triatomines. This study evaluated the systemic activity of three registered canine insecticides against *Triatoma infestans*, the principal domestic vector of Chagas disease in the Southern Cone of South America.

## Methods

### Selection of systemic anti-parasitic compounds

**Fig. 1:**
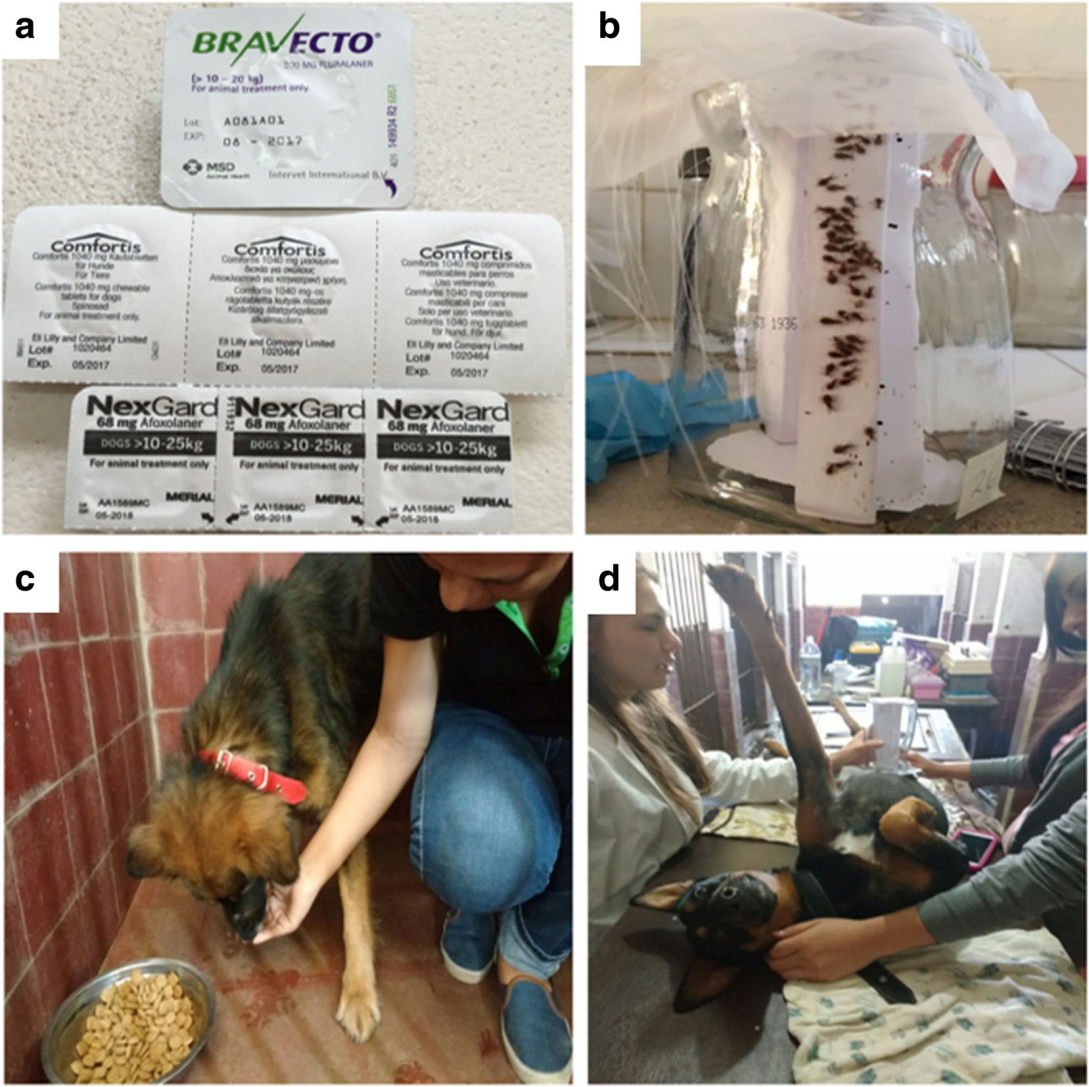
**a** Three candidate commercial insecticides under evaluation (Bravecto®, NexGard® and Comfortis®). **b** F1 generation triatomine colony established from intra-domicillary adult *T. infestans* collected in Itanambikua, Camiri. **c** Oral administration of drugs to recruited dogs. **d** Entomological bioassays, exposing second and third stage *T. infestans* instars to treated and control dogs for 30 min at 2, 7, 21, 34 and 51 days post-treatment

Three insecticide products registered for use in dogs in the USA/Europe and Bolivia were tested (Table 1), selected amongst other registered products based on the criteria that they (i) showed evidence of anti-ecto-parasite activity; (ii) were orally administered, and (iii) had an effective duration of at least 4 weeks, as described fully elsewhere [14]. The label claim of all three drugs is to combat fleas and ticks, with additional published studies showing varying activities against phlebotomine sandflies (vectors of *Leishmania* and some viruses) [12, 13]. All candidate products were administered in the recommended dose from the manufacturer (Table 1, Fig. 1a).

**Table 1:** Properties of candidate systemic anti-parasitic compounds for canine feed-through assays

Commercial name (Company)	Active compound (Family)	Dose (mg/kg)^a^	Mode of action	Safety (mg/kg)	Activity period^a^	Reference
Comfortis® (Elanco Animal Health)	Spinosad (Spinosyns)	45–70	Binds nicotinic acetylcholine receptors	Oral >3600	4 weeks	[30, 34, 40–43]
Bravecto® (Merck Animal Health)	Fluralaner (Isoxazolines)	25	Non-competitive GABA receptor antagonist	Oral >2000	12 weeks	[14, 18, 19, 27, 37, 44]
NexGard® (Merial)	Afoxolaner (Isoxazolines)	2.5	Non-competitive GABA receptor antagonist	Oral >1000	5 weeks	[24, 45–48]

^a^Registered dose for use in dogs against ticks and fleas [49–51]

### Dogs recruitment and maintenance

Dogs were obtained from the Zoonosis Surveillance and Control Program in the city of Santa Cruz de la Sierra, Bolivia. The Program routinely collected stray dogs, which were housed in municipal kennels for 3 days, followed by euthanasia if unclaimed by their owners. With agreement from the program, 12 unclaimed dogs were recruited. Included dogs weighed ≥5 kg and were outbred (mongrels). Dogs were transferred to purpose-built individual kennels (W × L × H: 0.97 × 1.30 × 1.70 m) at the Facultad de Ciencias Veterinarias, Universidad Autonóma Gabriel René Moreno (UAGRM), Santa Cruz. Dogs were acclimatized for 1 month prior to the experiment, where they were provided with regular nutrition and exercise. This period was sufficient time to minimize any unseen anti-parasitic treatment (e.g. ivermectin against *Dirofilaria* heart worm, or topical insecticides against fleas and ticks) that dogs may have received before their capture. The duration of the experiment was 51 days post-treatment. Dogs were routinely observed, wholesomely fed and watered, and exercised twice daily by veterinary staff. At the end of the experiment, all dogs were adopted, most by veterinary staff and students.

### Triatomine bug colony maintenance

An F1 generation triatomine colony was established in the Facultad de Ciencias Veterinarias (UAGRM) starting with eggs laid by intra-domicillary adult *T. infestans* collected in the endemic rural village of Itanambikua, Camiri, between December 2015 and March 2016 (Fig. 1b). The colony was established and amplified by blood-feeding nymphs and adults every 2–3 weeks on a group of 20 adult chickens, mildly restrained in a loose fitting three sided box enclosure, allowing free head movement, in a semi-darkened room, according to approved ethical protocols.

### Canine feed-through assays

Dogs were randomised into 3 treatment groups each group comprising 3 treated dogs and 1 untreated (control) dog. Randomisation was performed by tossing a coin in the presence of an observer. Following manufacturers’ recommendations, insecticides were administered on the same day as a single oral dose, one drug per treatment group (Fig. 1c). An entomological bioassay was performed on treated and control dogs starting 2 days after canine treatment, and at 7, 21, 34 and 51 days later. Bioassays were performed on all dogs on the same day. The 51-day period of follow-up is the equivalent of 7.3 weeks which exceeds the 4–5 week activity period of Nexgard® and Comfortis®, and represents over half (61%) of the 12 week activity period of Bravecto® (Table 1).

Colony reared second and third stage nymphs were used in the bioassays. Dogs were gently restrained by a trained veterinary student in a standing or lying down position to suit the dog’s comfort, and a glass jar with a net lid containing an average of 15.1 (range of 15–17) bugs starved for 2–3 weeks was placed on the dog’s abdomen and allowed to feed for 30 min (Fig. 1d). Half an hour was judged to be the time it took for a bug to fully engorge. Immediately after removing the jar, bugs were classified visually as fully engorged, semi-engorged, or non-fed. No bug deaths were observed at this time. Maintained in the same jar, the bugs were subsequently monitored at 24, 48, 72, 96 and 120 h post-exposure to record the numbers alive, dead or moribund.

### Statistical analysis

For purposes of analysis, only visibly engorged bugs were confirmed as exposed; non-fed bugs were considered unexposed. Moribund bugs were defined as those with movement impairment (i.e. limited activity and legs twitching or kicking in the air). The cumulative numbers of dead and moribund bugs per dog bioassay were computed for each of the five time-points post-canine treatment. The effect of the treatments on *T. infestans* mortality and morbidity were analysed separately. Data were analysed using General Linearised Models (GLM) for the appropriate Poisson, Binomial or Gausssian untransformed data structure, as indicated in the text. Unless specified, all analytical models included covariates describing the experimental structure [insecticide product, days post-canine treatment, dog status (control or treated)] and the interaction terms as appropriate. For example, to test for the effect of triatomine engorgement status on mortality estimates, the engorgement status × time post treatment interaction term was included in the model. Data were also analysed using specified non-parametric tests as required. In an analogy to an Intention to Treat analysis, we also quantified the efficacy of the drugs by including all triatomines in the bioassay jar offered to feed on treated dogs irrespective of the bugs’ feeding status outcome (i.e. fed or non-fed). All statistical analyses were conducted in Stata 13 (StataCorp LP, College Station, TX).

## Results

At the start of the experiment, recruited dogs weighed an average of 16.3 kg (SD: 5.93; range: 5–29 kg), with an estimated age of 5.9 yrs. (SD: 3, range: 2–12). There were no significant differences in the weights or ages of dogs between treatment groups (GLM: *Z* < 0.92, *P* > 0.36), or between control and treated dogs (GLM: *Z* < 0.39, *P* > 0.70). Furthermore, no adverse events among any dogs were observed during the study.

**Fig. 2:**
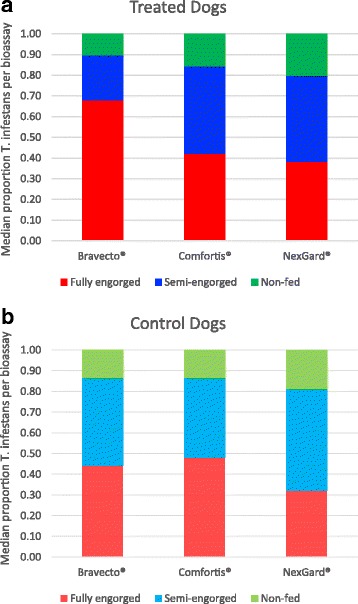
Overall feeding success of 907 colony-reared *T. infestans* immediately following bioassays (*n* = 5 bioassays per dog) on treated (**a**) and control (**b**) dogs

A total of 907 colony-reared *T. infestans* were offered to feed on the 12 recruited dogs in the 5 follow-up bioassays conducted from 2 to 51 days post-canine treatment. Of these bugs, 768 (84.7%) successfully blood-fed and were therefore classified hereafter as exposed, whereas 15.3% (139/907) did not feed so exposure was not confirmed, thus classified as non-exposed (Fig. 2, Table 2).

**Table 2:** Summary total numbers of colony-reared *T. infestans* exposed to dogs, and their feeding success, recorded immediately after bioassays (*n* = 5 bioassays per dog)

	Status of triatomines after bioassays				
Treatment group	Potentially exposed^a^	Blood-fed^b^	Fully engorged	Semi-engorged	Non-fed^c^
Treated (3 dogs each)					
Bravecto®	225	202	153	49	23
Comfortis®	230	194	97	97	36
NexGard®	227	181	87	94	46
Total	682	577	337	240	105
Control (1 dog each)					
Bravecto®	75	65	33	32	10
Comfortis®	75	65	36	29	10
NexGard®	75	61	24	37	14
Total	225	191	93	98	34
Overall total	907	768	430	338	139

^a^Numbers of bugs in the jar placed on the dog’s abdomen and given a choice to feed

^b^Classified as exposed

^c^Classified as unexposed

The per bioassay variation in the crude numbers of bugs exposed, and of different engorgement status (Table 3), was not dissimilar between treatment groups when controlling for the experimental design covariates [mean number of bugs per jar (potentially exposed): Poisson GLM *Z* < 1.93, *P* > 0.05; mean number of bugs engorged (exposed): Poisson GLM *Z* < -1.14, *P* > 0.25)]. The mean proportions of bugs that blood-fed or that fully engorged were not significantly different between treated or control dogs within drug treatment groups (Wilcoxon signed-rank tests: *Z* < 0.262, *P* > 0.793). However, the proportion of blood-fed bugs that fully (cf. partially) engorged on Bravecto® -treated dogs was higher than observed for NexGard® or Comfortis® (binomial GLM: *Z* > -3.52, *P* < 0.001); NexGard® or Comfortis® were not significantly different (*Z* = 1.55, *P* = 0.121). The proportion of bugs that blood-fed, or that were fully engorged, was not statistically associated with the number of bugs in the jar potentially exposed during the bioassay (GLM: test of number exposed: *Z* < 1.73, *P* > 0.08; test of drug*number exposed interaction terms: Z < 1.41, *P* > 0.16). This suggests that feeding success was not density-dependent.

**Table 3:** Variation in the median (binomial 95% CIs) number of bugs, and their proportional engorgement status, recorded immediately after each dog bioassay (*n* = 5 bioassays per dog)

Treatment group	Bravecto®	NexGard®	Comfortis®
Treated (3 dogs each)			
Total fed (exposed)	13 (13–15)	12 (9.36–14)	13 (12–14.82)
Total in jar (potentially exposed)	15 (15–15)	15 (15–15)	15 (15–15.82)
Proportion fed^a^	0.87 (0.87–1.00)	0.80 (0.62–0.93)	0.87 (0.8–0.94)
Proportion fully engorged^a^	0.67 (0.55–0.87)	0.33 (0.21–0.58)	0.40 (0.28–0.52)
Proportion of blood-fed that fully engorged	0.77 (0.67–0.87)	0.39 (0.24–0.73)	0.47 (0.34–0.65)
Control (1 dog each)			
Total fed (exposed)	14 (9–15)	15 (4–15)	14 (9–14)
Total in jar	15 (15–15)	15 (15–15)	15 (15–15)
Proportion fed^a^	0.93 (0.60–1.00)	1.00 (0.27–1.00)	0.93 (0.60–0.93)
Proportion fully engorged^a^	0.40 (0–0.80)	0.33 (0–0.60)	0.33 (0.07–0.93)
Proportion of blood-fed that fully engorged	0.44 (0–0.86)	0.50 (0–0.60)	0.56 (0.07–1.00)

^a^Proportion of all bugs offered to dogs in the bioassay (i.e. potentially exposed)

### Treatment lethality effect

**Fig. 3:**
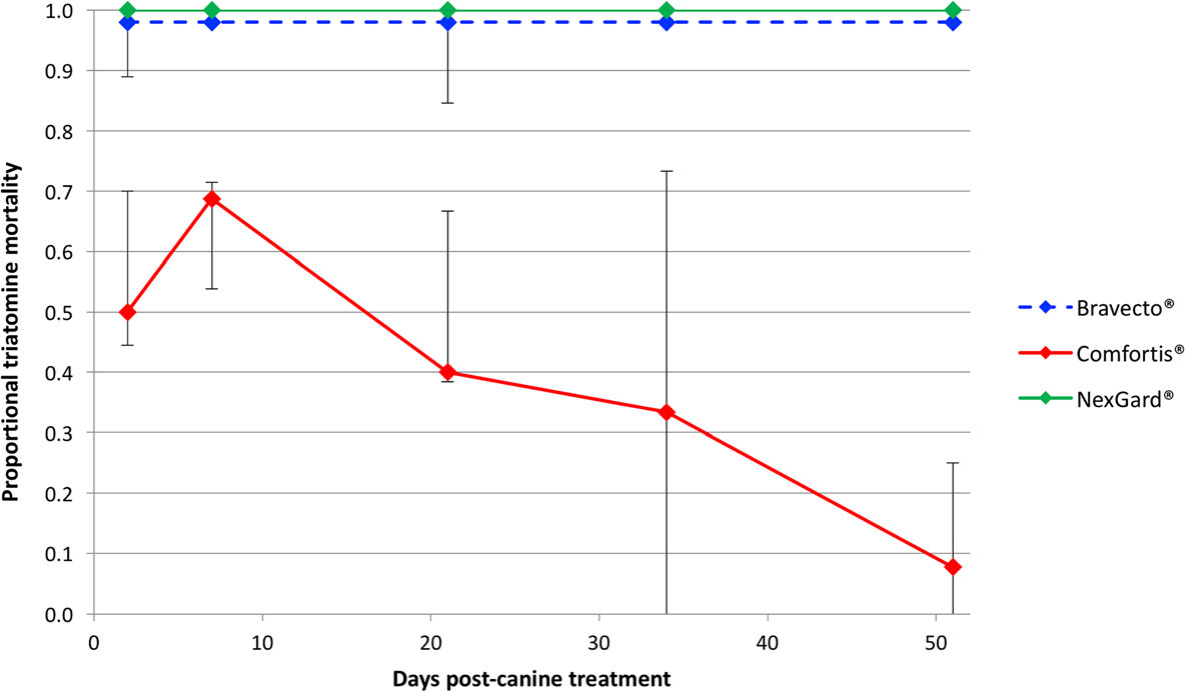
Proportional mortality of bugs exposed to each drug in post-canine treatment bioassays. Mortality in control dogs were zero except 1 death in the Comfortis group® (data not shown; see Table 4). Data are medians and binomial estimates of 95% CIs (error bars). Bravecto® and NexGard® displayed 100% mortality in treated dogs but is offset for clarity

Bravecto® and NexGard® induced high levels of *T. infestans* mortality. The proportion of fed bugs (fully- and semi-engorged combined) that died by 120 h post-exposure was 100% in all follow-up bioassays following canine treatment (Table 4, Fig. 3). The lethality effect in dogs treated with Comfortis® reached a median of 50–70% within 7 days post-canine treatment, but declined linearly thereafter (Fig. 3). Engorgement status did not influence the high mortality rates in Bravecto® and NexGard® treated dogs, whereas there was some evidence that for Comfortis®, mortality was higher in fully-engorged (median 0.64, binomial 95% CI: 0.14–0.98, *n* = 15) compared to semi-engorged bugs (median 0.23, binomial 95% CI: 0.00–0.63, *n* = 14) (Wilcoxon signed-rank test of matched proportional mortality per bioassay sample post canine treatment: *Z* = 1.74, *P* = 0.082; crude totals 0.50 (48/97) *vs* 0.32 (31/97) (*χ* ^2^ = 6.17, *P* = 0.013).

**Table 4:** Summary of mortality amongst exposed and non-exposed *T. infestans* post-canine treatment bioassays^a^

Days post-canine treatment	Triatomines applied to treated dogs		Triatomines applied to control dogs	
	Proportion (dead/exposed)	Dead/Non-exposed	Dead/Exposed	Dead/Non-exposed
Bravecto®				
2	1 (42/42)	0/3	0/9	0/6
7	1 (41/41)	1/4	0/14	0/1
21	1 (43/43)	0/2	0/13	1/2
34	1 (36/36)	1/9	0/14	0/1
51	1 (40/40)	1/5	0/15	0/0
Total	1 (202/202)	0.13 (3/23)	0.0 (0/65)	0.10 (1/10)
Comfortis®				
2	0.55 (16/29)	0/17	0/14	0/1
7	0.63 (27/43)	1/4	0/9	0/6
21	0.48 (19/40)	0/5	0/14	0/1
34	0.36 (16/44)	0/2	0/14	0/1
51	0.03 (1/38)	3/8	1/14	0/1
Total	0.41 (79/194)	0.11 (4/36)	0.02 (1/65)	0.0 (0/10)
NexGard®				
2	0.97 (29/30)	1/17	0/4	0/11
7	1 (32/32)	0/13	0/12	0/3
21	0.95 (38/40)	0/5	0/15	0/0
34	1 (39/39)	4/6	0/15	0/0
51	1 (40/40)	0/5	0/15	0/0
Total	0.98 (178/181)	0.11 (5/46)	0.0 (0/61)	0.0 (0/14)
Total (3 treatments)		0.11 (12/105)	0.01 (1/191)	0.03 (1/34)

^a^Cumulative numbers dead by 120 h post-triatomine exposure/potential exposure

Three measures of background mortality were obtained. The mortality in triatomines that engorged on control dogs was zero for all drugs with the exception of 1/14 bugs exposed to Comfortis® on day 51 post-canine treatment (Table 4). Similarly, mortality amongst non-exposed (unfed) bugs offered to control dogs was zero with the exception of 1/2 bugs offered to the Bravecto® control dog 21-days post-canine treatment. The third measure was for unexposed bugs in the treated dog groups, amounting to 11.4% (12/105) across treatments, with no significant differences detected between drug treatments (Bravecto® 3/23, Comfortis® 4/36, NexGard® 5/46; *χ* ^2^ < 0.07, *P* > 0.79 for each paired test). By comparison, mortality in the non-exposed bugs on all control dogs was 2.9% (1/34), which was not statistically different (Fisher’s exact test, *P* = 0.30) (Table 4). However, crude mortality in non-exposed bugs on treated dogs was higher than in exposed bugs on control dogs 0.52% (1/191) (Fisher’s exact text, *P* < 0.001) suggesting that a proportion of non-exposed bugs on treated dogs were misclassified and were actually partially fed.

### Treatment morbidity effect

**Fig. 4:**
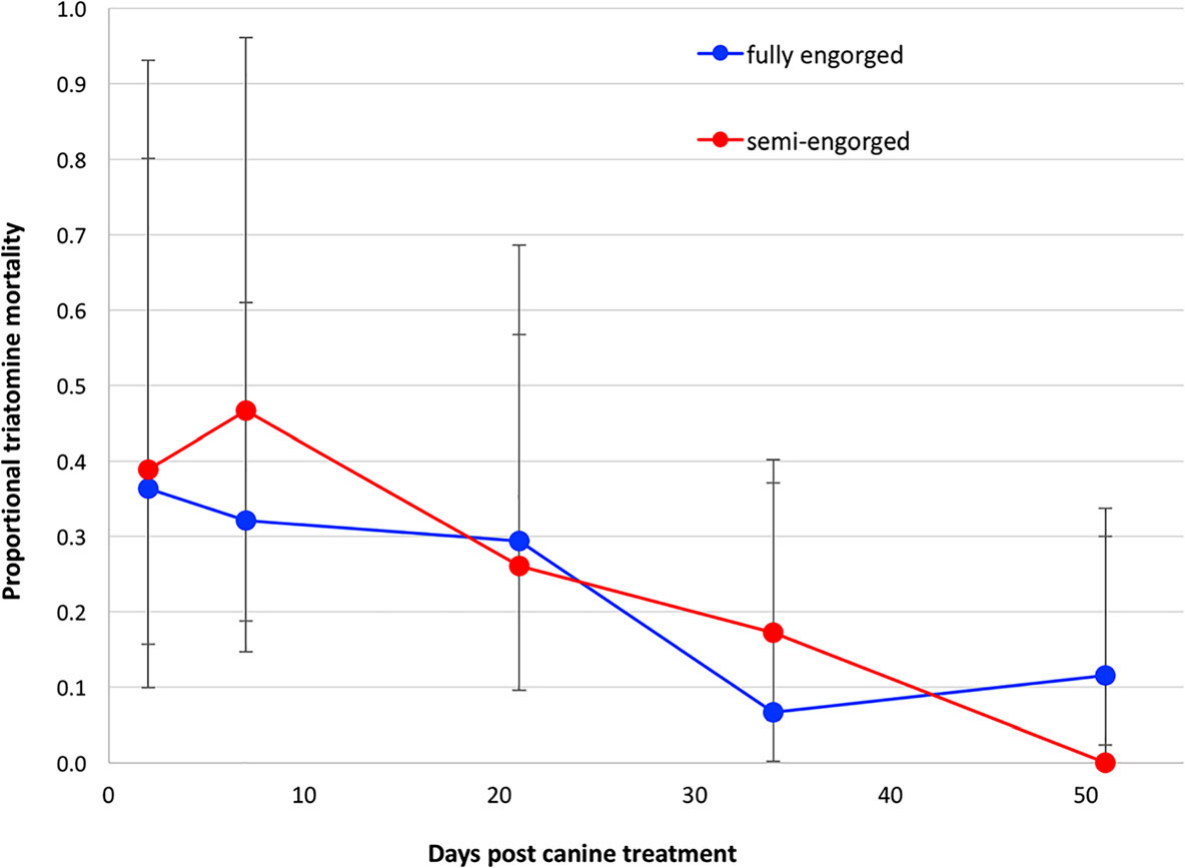
Morbidity of fully- and semi-engorged bugs exposed to Comfortis® post canine treatment. The 95% CIs represent the variation between individual dogs

The three drug treatments induced morbidity, defined as movement impairment post-exposure, in a varying proportion of engorged bugs, and with different progression outcomes. Morbidity induced by Comfortis® treatment was observed in a cumulative 47/194 (24%) exposed bugs in all five follow-up bioassays and on all 3 treated dogs (Table 5). Bravecto® induced morbidity in only 3% (6/202) of exposed bugs, and NexGard® in 13% (24/181) of exposed bugs. The proportions of bugs that showed signs of morbidity were significantly higher following exposure to Comfortis® than to the other treatments (*χ* ^2^ > 10.7, *P* < 0.0012 in paired comparisons; Kruskal-Wallis equality-of-populations rank test *χ* ^2^ = 13.2, *P* = 0.0014), but not different between NexGard® and Bravecto® treatments (Kruskal-Wallis *χ* ^2^ = 1.0, *P* = 0.31). Considering Comfortis® alone, the proportion of moribund exposed bugs significantly declined with increasing time from canine treatment (Wilcoxon rank-sum test for trend: *Z* = -2.40, *P* = 0.016). Similar patterns were seen in fully- and semi-engorged bugs (comparison of days post-treatment*engorgement status interaction, GLM binomial, *Z* = -0.73, *P* = 0.46) (Table 5, Fig. 4).

**Table 5:** Summary of proportion morbid *T. infestans* in the Comfortis® treatment group (*n* = 5 bioassays per dog)

Days post-canine treatment	Treated dogs				Control dogs
	Exposed^a^	Fully engorged	Partially engorged	Non-exposed^b^	Non-exposed and exposed
2	0.38 (11/29)	0.36 (4/11)	0.39 (7/18)	0 (0/17)	0 (0/15)
7	0.37 (16/43)	0.32 (9/28)	0.47 (7/15)	0 (0/4)	0 (0/15)
21	0.28 (11/40)	0.29 (5/17)	0.26 (6/23)	0 (0/5)	0 (0/15)
34	0.14 (6/44)	0.07 (1/15)	0.17 (5/29)	0 (0/2)	0 (0/15)
51	0.08 (3/38)	0.12 (3/26)	0.0 (0/12)	0 (0/8)	0 (0/15)
Total	0.24 (47/194)	0.23 (22/97)	0.26 (25/97)	0.0 (0/36)	0.0 (0/75)

^a^Blood-fed triatomines were classified as exposed

^b^Non-engorged triatomines classified as non-exposed

Both Bravecto® and NexGard® induced morbidity in the 51-days post-treatment bioassay only; Bravecto® affected 3 semi- and 3 fully-engorged bugs in bioassays on 2 dogs, whereas NexGard® affected 2 partially and 22 fully-fed bugs in bioassays on the 3 dogs. However, these treatments were not considered sub-lethal doses as 100% (Bravecto®: 6/6; NexGard®: 24/24) of these bugs died within 48 h after feeding (Table 6).

**Table 6:** Fate of initially morbid bugs post-exposure

Treatment	Initial proportion (morbid/exposed)	Fate of morbid bugs		Proportion (number/initially morbid)^a^
		Remained morbid	Dead	Recovered
Bravecto®	0.03 (6/202)	0 (0/6)	1 (6/6)	0 (0/6)
NexGard®	0.13 (24/181)	0 (0/24)	1 (24/24)	0 (0 /24)
Comfortis®	0.24 (47/194)	0.53 (25/47)	0.17 (8/47)	0.30 (14/47)

^a^Fate recorded as outcome after 120 h follow-up observation period post-exposure

In contrast, morbidity induced by Comfortis® treatment was observed in all 5 follow-up bioassays on the 3 treated dogs (Table 6). Of the 47 morbid exposed bugs, only 17% (8/47) were dead by the end of the 120 h post-exposure; 4 died within 48 h of feeding, and 4 delayed to 72 and 120 h post-feeding, respectively. Morbidity persisted in 53% (25/47) of these bugs by the end of the 120 h observation period, whereas 30% (14/47) appeared to have recovered to full activity within this time frame (Table 6).

No morbidity was observed among the non-exposed bugs on Comfortis® treated dogs, bugs on Comfortis® control dogs, or bugs exposed or non-exposed on Bravecto® control dogs (Table 5). Only 1 non-exposed bug on the NexGard® control dog showed signs of morbidity.

### Impact of morbidity on triatomine feeding success

To test if moribund *T. infestans* could successfully feed, and thus be potentially infectious, 8 partially-fed moribund bugs and 7 active control bugs that had been exposed to 3 different dogs 21 days post-canine treatment with Comfortis®, were subsequently exposed to a new, non-treated dog 30 days post-bug exposure (at the 51 day follow-up). None of the moribund bugs attempted to feed (0/2, 0/1 and 0/5 per dog), whereas 6/7 (86%; 1/1, 2/3 and 3/3) of the control bugs attempted and successfully fed.

### Intention to treat analysis

In an “Intention to Treat” analysis, the efficacy of the drugs to kill *T. infestans* was 91% (95% CI: 79.1–100%), 81% (95% CI: 69.4–93.2%) and 36% (95% CI: 28.7–44.7%) for Bravecto®, NexGard®, and Comfortis®, respectively. The equivalent analysis for induced morbidity were 3% (95% CI: 1.0–5.8%), 11% (95% CI: 7.1–16.3%), and 20% (95% CI: 15.0–27.2%) for the three drugs. Out of 75 bugs offered to each control dog, only 1 died in each of the Bravecto® and Comfortis® treatment groups, and no bugs showed signs of morbidity in any treatment group.

Analysis of the odds ratios associated with drug induced mortality showed that in comparison to Bravecto® (referent), the odds of mortality from NexGard® were 0.70 (95% CI: 0.502–0.982) and from Comfortis® 0.17 (95% CI: 0.122–0.246). The odds of inducing morbidity compared to Bravecto® were 8.9 (95% CI: 3.76–21.22) and 4.4 (95% CI: 1.79–10.94) for NexGard® and Comfortis®, respectively.

## Discussion

Of the three candidate drugs under evaluation, Bravecto® and NexGard® demonstrated the most promising results. Both compounds induced and sustained high levels of triatomine mortality, killing all bugs within 120 h of feeding for 51 days post-canine treatment. By contrast, the initial lethality effect of Comfortis® was much lower and had declined almost entirely by the end of the experiment. While mortality was the primary outcome of Bravecto® and NexGard® exposure, Comfortis® treatment resulted in substantial morbidity. One third of these initially moribund bugs fully recovered within five days post-exposure, whereas over half remained in a morbid state for at least five days post-exposure, but were subsequently unable to feed even 30 days later. In addition to its efficacy and residual activity, Bravecto® also displayed two other advantageous characteristics; significantly more fed bugs took complete blood meals, potentially maximizing their likelihood of receiving a lethal dose especially once canine plasma levels begin to fall, and exposure produced more immediate knockdown, compared to the other two drugs. Why higher proportions of bugs fully-engorged on Bravecto® treated dogs than the other two drugs, which was consistent from time of treatment, is not known; the data do not suggest an anti-feeding effect of the other systemic insecticides. Accounting for this difference in an Intention to Treat analysis, Bravecto® proved to induce higher mortality and lower morbidity.

The generally analogous results demonstrated by Bravecto® and NexGard® can be explained by their similar mechanistic pathways. Fluralaner and Afoxolaner are members of the isoxazoline family and potent antagonists of γ-aminobutyric acid (GABA)- and L-glutamate-gated chloride channels, resulting in irreversible hyper-excitation, with significant selectivity for arthropod *vs* mammalian neurons [15]. Both compounds have good mammalian safety profiles and are highly efficacious against medically-important vectors and other arthropods [14, 16, 17]. A single dose of Bravecto® achieved >99% mortality of canine fleas, *Rhipicephalus, Ixodes* and *Dermacentor* ticks and *Demodex* mites, over 12 weeks, with measurable plasma concentrations detected up to 112 days post canine-treatment; mean half-life was estimated at 15 days [18–20]. Fluralaner has also outperformed a number of other topical insecticides and systemic insecticides, including fipronil, sarolaner, imidacloprid and permethrin against *Rhipicephalus sanguineus* and dieldrin to control *Ctenocephalides felis, Aedes aegypti* and *Lucilia cuprina* [15, 21]. Similarly, a single dose of NexGard®, reduced populations of *C. felis* by 100% within 24 h and blocked egg production by >99% over 36 days [22, 23]; additional re-treatment at days 28–30, achieved total flea control by 60 days [24]. Other studies of single doses of NexGard® report complete elimination of pre-existing *Dermacentor reticulatus, D. variabilis* and *Ixodes ricinus* infestations and sustained efficacy of >96% one month later [25, 26] and significant reductions in *R. sanguineus* and *Haemaphysalis longicornis* by >96% after 35 days [27] and 92% after 30 days [28], respectively; maximum plasma levels are observed at 2–6 h and mean half-life is 15.5 days [17]. A direct comparison of immediate efficacy of Bravecto® and NexGard® against *R. sanguineus* demonstrated that onset of activity was 4 h and 8 h, respectively, and 100% mortality was achieved within 12 h and 48 h, respectively [21].

By comparison, Comfortis® is a mixture of two spinosyns (spinosyn A and D), which preferentially binds to nicotinic acetylcholine receptors (nAChRs), with secondary activity against GABA-receptors [29]. This drug can also be a potent pulicide, reducing *C. felis* burdens by >99 and 97–98% three and four weeks post-treatment in laboratory infestations, respectively, and by >99% after a 90 day multi-country community trial [30]. Other investigators have presented more variable residual activities against fleas over 28 days [31, 32]. In contrast to our results, previous studies have reported complete knockdown of established flea populations [33] and 100% mortality within 4 h [31], coinciding with peak canine spinosad plasma levels, between 2 to 4 h post-treatment [30]. Spinosyns have also demonstrated high levels of efficacy against *Phlebotomus papatasi* feeding on treated hamsters (5000 mg/kg), producing 100% sand fly mortality for one week; mortality declined to 14.2% by day 14 [34]. In addition to triatomines likely requiring higher concentrations of the active ingredient than fleas and smaller arthropods, other possible explanations for our observations include that a lower proportion of triatomines became fully-engorged following Comfortis® exposure, compared to those feeding on Bravecto®-treated dogs, and/or systemic concentrations had already begun to fall by the first time-point; mean elimination half-life for spinosad ranges from 128 to 163 h [35]. Because Comfortis® elicited the lowest levels of triatomine mortality, had the shortest residual activity, and given reports of the potential presence of naturally-resistant triatomine populations in neighbouring Argentina [36], this compound does not currently warrant further consideration for control of *T. infestans* populations in Bolivia. Notwithstanding, screening of a wide range of drugs with different modes of action for potential rotation in a community-setting, remains important to reduce the risk of natural and acquired resistance.

When evaluating oral insecticides to develop into a community-level control strategy, compounds need to demonstrate high and sustained anti-triatomine efficacy, long residual activity to reduce re-treatment times, be administrable to a large number of dogs, safe in case one dog receives multiple doses, and provide additional benefits, such promoting improved nutritional status and control of other ecto- and endo-parasites. In this regard, Bravecto® emerges as the optimal candidate product based on its high levels of knockdown, mortality and longest reported residual activity. Furthermore, ingestion of Bravecto® with food was previously shown to increase total drug exposure over 91 days and was 2.5 times greater than in dogs treated while fasting [37].

The aim of the current study was to demonstrate proof of principle of the systemic lethality of candidate oral insecticides against triatomine bugs and thus there are some limitations which must be considered when interpreting the results. For logistical reasons, the study was not blinded to the personnel handling the dogs. The background mortality in unexposed bugs on treated dogs across treatments was significantly higher than in exposed bugs on control dogs, but not different for non-exposed bugs on control dogs. The former result raises the possibility that a proportion of the bugs on treated dogs were misclassified as non-fed (non-exposed) via visual inspection rather than by microscopic examination to detect small amounts of blood. We necessarily reduced handling of triatomines in this study to minimize any experimental mortality due to the fragility of the smaller instars. However, this does not appear to have introduced any discernable bias as the three drugs demonstrated consistent mortality rates amongst exposed bugs. While we did not expect to observe dramatic differences in drug efficacy between different development stages, susceptibility to other insecticides is reported to be more variable [38, 39]. In this study, we elected to evaluate second and third instars for operational reasons and for logistics of colony production. In a community scenario, all five triatomine stages are capable of harbouring *T. cruzi* but younger, immature bugs must take additional blood meals to develop, providing more opportunities for *T. cruzi* transmission; these instars would also likely be killed by lower insecticide concentrations and therefore are the ideal life-cycle stage to target. We also chose to use outbred, local dogs, which ranged in terms of age, weight and probably micronutritional status, again to simulate as true to life results as possible. This study was conducted for 7.3 weeks as a compromise between the predicted activity periods of all three candidate drugs. By the final time-point Bravecto® and NexGard®, which were assumed to remain efficacious for at least 12 and 5 weeks, respectively, were still killing all exposed bugs. Future studies with longer follow-up times are required to determine the residual duration of these two compounds and their rate of decline, complemented by assays measuring both canine plasma levels and concentrations of active ingredients imbibed by triatomines. Furthermore, this experimental design could be expanded to establish the minimum effective doses for *T. infestans* to inform the frequency of re-treatment required in a community setting and to investigate the impact of sub-lethal doses on triatomine morbidity, longevity and fecundity.

## Conclusion

A single oral dose of Bravecto® or NexGard®, two isoxazoline compounds, commercially available and registered for tick and flea control in dogs and cats, were safe and well tolerated, producing 100% triatomine mortality within 5 days of feeding on treated dogs over 7.3 weeks. While both drugs were highly efficacious, more triatomines exposed to Bravecto® were immediately knocked-down and took complete blood-meals, potentially maximizing their likelihood of receiving a lethal dose. Coupled with the likely longer residual activity of Bravecto® compared to NexGard® (12 *vs* 5 weeks, respectively), to reduce re-treatment times and to address issues of longer-term sustainability, Bravecto® represents an ideal candidate systemic insecticide for development into a complementary, operationally-feasible, community-level method of reducing triatomine infestation and potentially interrupting *T. cruzi* transmission. Notwithstanding the current cost of Bravecto® being somewhat higher than that of NexGard®, a strategy to apply an oral veterinary drug to combat a public health problem would be suited to both hyperendemic regions, such as the Gran Chaco, and low endemic regions e.g. Costa Rica, where dogs remain an important reservoir for onward transmission.
